# Texture analysis of magnetic resonance images of the human placenta throughout gestation: A feasibility study

**DOI:** 10.1371/journal.pone.0211060

**Published:** 2019-01-22

**Authors:** Quyen N. Do, Matthew A. Lewis, Ananth J. Madhuranthakam, Yin Xi, April A. Bailey, Robert E. Lenkinski, Diane M. Twickler

**Affiliations:** 1 Department of Radiology, UT Southwestern Medical Center, Dallas, Texas, United States of America; 2 Advanced Imaging Research Center, UT Southwestern Medical Center, Dallas, Texas, United States of America; 3 Department of Clinical Science, UT Southwestern Medical Center, Dallas, Texas, United States of America; 4 Obstetrics & Gynecology, UT Southwestern Medical Center, Dallas, Texas, United States of America; Northwestern University Feinberg School of Medicine, UNITED STATES

## Abstract

As fetal gestational age increases, other modalities such as ultrasound have demonstrated increased levels of heterogeneity in the normal placenta. In this study, we introduce and apply ROI-based texture analysis to a retrospective fetal MRI database to characterize the second-order statistics of placenta and to evaluate the relationship between heterogeneity and gestational age. Positive correlations were observed for several Haralick texture metrics derived from fetal-brain specific T2-weighted and gravid uterus T1-weighted and T2-weighted images, confirming a quantitative increase in placental heterogeneity with gestational age. Our study shows the importance of identifying baseline MR textural changes at certain gestational ages from which placental diseased states may be compared. Specifically, when evaluating for placental invasion or insufficiency, findings should be evaluated in the context of the normal placental aging process, which occurs throughout gestation.

## Introduction

The human placenta is a complex structure with unique capabilities. It has a 40-week average life span during which it facilitates the exchange between the maternal and fetal cardiovascular systems [1]. The placenta undergoes extensive growth and remodeling through trophotrophism and maturation of the maternal-fetal units within the intervillous spaces of the cotyledons. These events have been extensively studied through *in vitro* assessments and pathologic evaluation [2]. However, the development and maturation of the human placenta *during pregnancy* is relatively unknown and quantitative studies describing growth and aging characteristics of the placenta throughout the gestational period are lacking [3]. This is an important area of research because abnormal placental growth and function are related to serious conditions such as preeclampsia, gestational diabetes, preterm labor and birth, and stillbirth. In addition, the high-risk clinical scenario of invasion and the morbidly adherent placenta in women with previous cesarean delivery has become increasingly prevalent, and imaging plays a significant role in its detection [4,5].

The placenta becomes significantly more heterogeneous with fetal gestational aging, both through the maturation of the cotyledons and other processes associated with aging, such as fibrin accumulation and calcification [6]. The degree of placental maturity has historically been assessed by an ultrasound placental grading system (Grannum classification) [7]. Magnetic resonance imaging (MRI) has become a powerful diagnostic tool, offering excellent soft-tissue contrast, large field-of-view, and the ability to enhance different tissue types or biological function using various acquisition protocols. The MR imaging techniques and protocols have advanced to generate fetal images with unprecedented image quality [8,9], and improvements will continue as 3T imaging of the gravid female becomes increasingly more routine [10–12].

When abnormalities are observed initially with sonography, fetal MR imaging has been incorporated into routine clinical practice to answer specific questions, because of its superior spatial resolution of the fetus and the placenta. At our institution, 5–10 MR fetal imaging studies are performed on average per week to evaluate suspected fetal abnormalities. These studies include routine T2 weighted (T2W) and T1 weighted (T1W) imaging of the entire gravid uterus including the fetus and the placenta, followed by dedicated imaging to characterize the potential underlying anomalies. Each MRI study to evaluate fetal brain for suspected ventriculomegaly will include T2W and T1W imaging of the entire gravid uterus, followed by orthogonal fetal brain acquisitions with T2W, T1W, and diffusion-weighted (DW) images. Often, these dedicated imaging techniques include the placenta in the imaging field of view. Such fetal MR studies performed across pregnancy provide a potentially rich and untapped dataset of placental structure and function. Mild abnormalities of the fetal central nervous system presently are not known to affect placental pathology.

**Fig 1:**
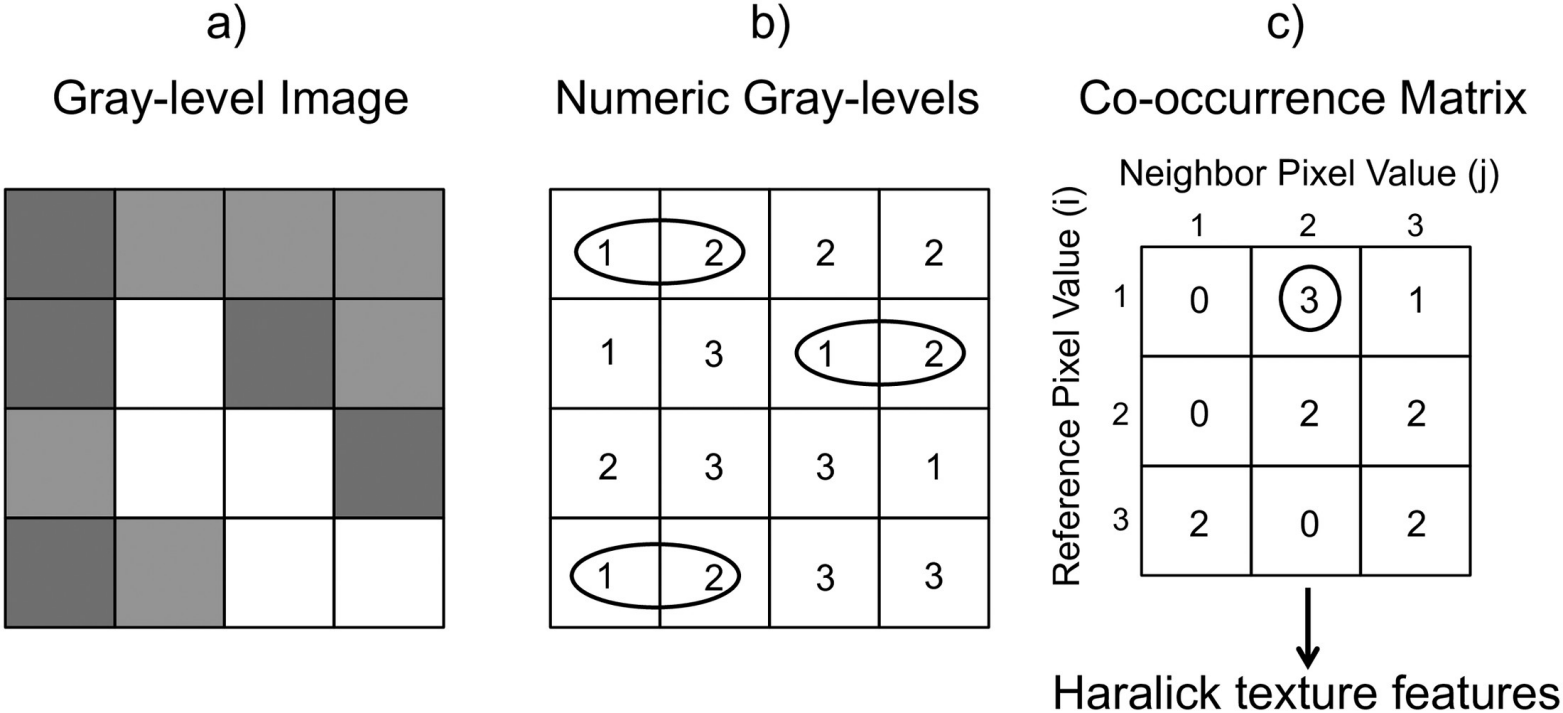
Haralick texture features are calculated from the gray-level co-occurrence matrix (GLCM). Example of how the GLCM is calculated for a given 4x4 pixel image (a) with the corresponding numerical gray-level pixel intensities (b) is shown here. GLCM is computed by going in horizontal direction with one pixel separation and recording the number of occurrences in which a pixel intensity of 2 is horizontally next to a 1 and allocated in the co-occurrence matrix (c). Figure adapted from ref. [20].

In recent years, structural and statistical analyses have been applied more routinely in medical imaging, particularly in the context of computer-aided diagnosis [13]. The Haralick texture analysis technique has been used for the evaluation of image texture, or the spatial arrangement of image patterns that provide the visual appearance of texture (smoothness, coarseness, etc.) in satellite imagery and aerial photography applications [14]. Recent applications of texture analysis in medical MR imaging have been in the evaluation of T2W, DWI, and DWI-derived apparent diffusion coefficient (ADC) images for multiple cancer evaluations [15–19]. It has been shown that texture analysis, through quantification of gray-level patterns and pixel inter-relationships within an image, is sensitive to tissue heterogeneity and potentially can aid in detection, diagnosis and tumor treatment response. The Haralick texture technique works by evaluating the spatial relationships between neighboring image pixels with second-order statistics, computing resultant gray-level co-occurrence matrix (GLCM), and calculating GLCM-derived textural features for each image (Fig 1) [20]. By comparing changes in texture feature values with specific parameter, relationship between these features (Table 1) and parameters can be evaluated. Texture features can also be used as an indication of image (or region of interest (ROI)) contrast or heterogeneity (i.e. entropy, which describes how the gray-level is distributed).

**Table 1:** The 13 Haralick texture features [14] that were calculated using the Python-based in-house developed software, pyOsirix, which was integrated into the DICOM viewer, Osirix.

Abbreviation	Haralick texture feature
f1	Angular second moment
f2	Contrast
f3	Correlation
f4	Sum of squares
f5	Inverse difference moment
f6	Sum average
f7	Sum variance
f8	Sum entropy
f9	Entropy
f10	Difference variance
f11	Difference entropy
f12	Information measures of correlation
f13	Information measures of correlation

Thus, the purpose of our study was two fold. First, we introduce and assess the feasibility of applying textural analysis on placenta ROIs from the standard gray-scale clinical fetal MR images. In order to distinguish between normal placental aging from pathology, the understanding of placental textural changes with gestational age is important. For our second aim, we characterize the placenta as a function of fetal gestational age based on the textural properties, and compare the results with the existing information that placenta heterogeneity increases with fetal aging.

A preliminary report of this work was presented in an abstract form at the 25^th^ annual meeting of the International Society for Magnetic Resonance in Medicine (ISMRM) [21].

## Materials and methods

### Imaging dataset

**Fig 2:**
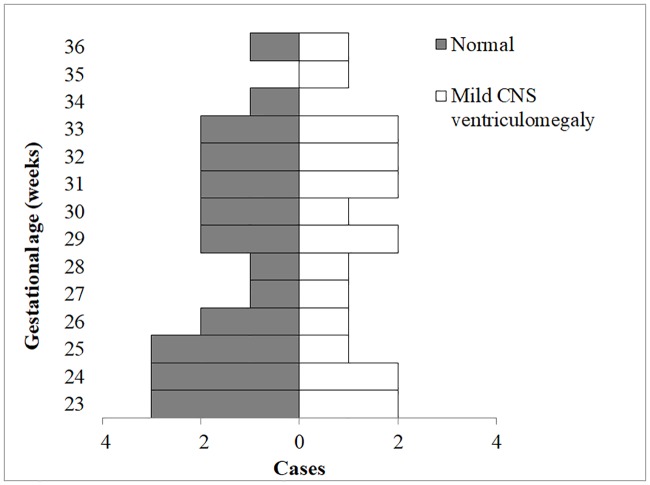
Demographics of the imaging cohort included 25 cases determined to be normal and 19 cases with mild central nervous system (CNS) ventriculomegaly based on MR findings. The gestational age ranged from 23 to 36 weeks.

The UT Southwestern Institutional Review Board approved this study. The approval number is STU 022016–018: Determining the MRI Features and Diffusion Characteristics of Normal Placenta and Placental Invasion. Written and informed consent were obtained from all participants. Fetal MRI examinations including DWI of the placenta and fetal brain were retrieved from our database spanning from 2006–2016. Written informed consents were obtained before all fetal MRI studies, and no sedation was administered during the examinations. The retrospective study was approved by the Institutional Review Board (IRB). Studies with extreme fetal movements resulting in MR artifacts were excluded from the dataset. For our initial evaluation, forty-four fetal imaging studies were selected from our MR database with the following inclusion criteria: singleton pregnancy, gestational age ranging from 23 to 36 weeks, normal or mild ventriculomegaly MR finding, MR acquisitions included T2W, T1W, DWI, and ADC with substantial inclusion of the placenta in the imaging field of view. A board-certified radiologist with 30 years of obstetric and gynecologic ultrasound and MR experience curated the dataset based on the described criteria. Gestational age was based on the current practice of last menstrual history and ultrasound dating. Of the forty-four cases, twenty-five were determined to be normal and nineteen had mild ventriculomegaly of the central nervous system. Ventriculomegaly was defined as atrial measurement greater than 10 mm in the axial MR plane of the fetal brain [22]. Characteristics of the study population are described in Fig 2.

All imaging data were acquired on a 1.5T MRI scanner (Avanto, Siemens Healthcare, Erlangen, Germany) with a body array coil for signal reception. Subjects were positioned in the lateral decubitus position, and all imaging was performed with maternal free breathing, except for fetal brain specific T1W acquisition. All subjects received T1W, T2W acquisitions capturing the gravid uterus including the fetus and the entire placenta, followed by fetal brain specific acquisitions with substantial inclusion of the placenta in the imaging FOV of T2W, T1W (breath-hold), and DWI using two b-values (b = 0, 800 s/mm^2^). Parameters for the T2W, T1W, and DWI sequences are described in Table 2. The ADC maps were automatically generated from the DWI series using the vendor provided tool on the main MR console.

**Table 2:** MRI parameters of the standard T2W, T1W, and DWI.

Acquisition	Sequence	TR (msec)	TE (msec)	Slice Thickness (mm)	Matrix size	Resolution
T1	FLASH	112–128	4.8	5–7	256x256	1.17x1.17
T2	HASTE	1100	77–84	5–7	256x256	1.17x1.17
DWI	DW-EPI	5100–8600	104	5	192x192	1.56x1.56

### Placenta segmentation and data analysis

**Fig 3:**
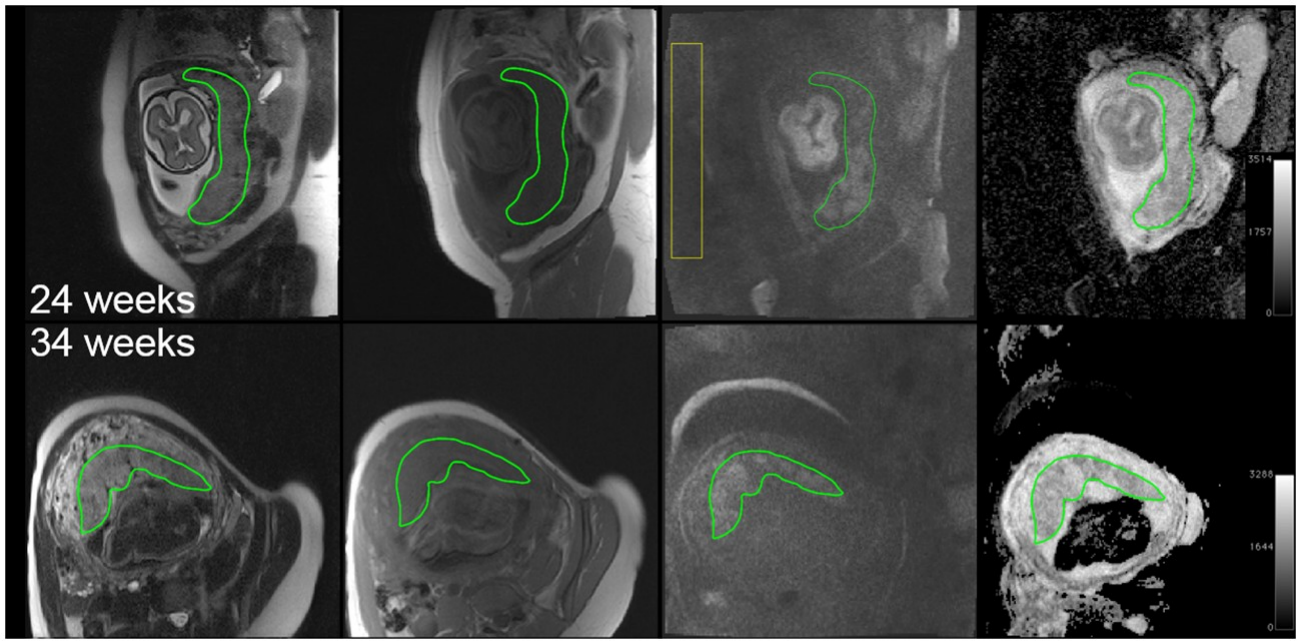
Left to Right: Representative T2W, T1W, DWI (b = 800 s/mm^2^), and ADC map of a 24-week (top row) and a 34-week (bottom row) gestation with their respective placental ROIs (green). For DW images SNR calculation, the image background noise ROI (yellow) is drawn outside of the tissue. The 24-week fetus was identified to have mild CNS ventriculomegaly; the 34-week fetus had normal MR findings.

All MR images were archived to the Radiology Research PACS system (iPACS, inVicro, Boston, Massachusetts) and then retrieved using a DICOM viewer (OsiriX version 5.8.2, Pimeo SARL, Bernex, Switzerland) running on Mac OSX for further processing. Volumetric placental ROIs were drawn on the T1W and T2W series covering the gravid uterus. For fetal brain specific acquisitions, single slice placental ROIs were drawn; the image had to include a large area of placenta within the uterine area as seen in Fig 3. All the ROIs were manually drawn by one researcher and confirmed by a board-certified radiologist (Fig 3). Placental volume and placental mean ADC values were extracted using OsiriX.

In order to evaluate the SNR of the DW images, signal intensity values of the placental region and area outside of the tissues (image background) were taken (Fig 3). The SNR of the DW images at the two b values were calculated using the following equation [23]:

$$SNR=0.655\times \frac{Placental\;Signal}{Standard\;deviation\;of\;the\;background\;noise}$$ (1)

The average value of SNR over a cohort of five subjects was computed to assess the reliability of DWI-derived ADC maps. SNR of T1W and T2W images were computed similarly in a cohort of five different subjects.

Python code for an Osirix-based plugin (pyOsirix) was developed to extract 13 Haralick texture features (Table 1) using the Mahotas library from manually segmented placental ROIs [24,25]. These features were calculated from the GLCM, with a spatial relationship defined as: distances = 1, 2; orientations = 0°, 45°, 90°, 135°. All metrics were exported to a CSV file for offline statistical analysis. Average value of the four angle GLCM was calculated. Due to image quality variation between the generated ADC maps, texture metrics from DWI and ADC series were calculated both with and without intensity normalization and histogram equalization [26]. Intensity normalization and histogram equalization have been described as appropriate pre-processing steps for minimizing inter-subject variance due to scanner parameters, as opposed to biological variance [27]. In compliance with a recent radiomics standardization initiative [28], our second order statistics analysis process can be summarized as the following: the ROI was analyzed slice by slice (2D), gray level co-occurrence matrix was extracted, and histogram equalization was used for image intensities discretization.

### Statistical analysis

Correlations between extracted Haralick texture features and gestational age as well as comparisons between normal and mild ventriculomegaly cases were investigated using Spearman’s correlation coefficient (ρ). False discovery rate (FDR) adjusted p values (q values) were calculated. The q values less than 0.05 were considered statistically significant. The statistical analysis was performed using SAS software (Version 9.4, SAS Institute Inc., Cary, NC).

## Results

**Fig 4:**
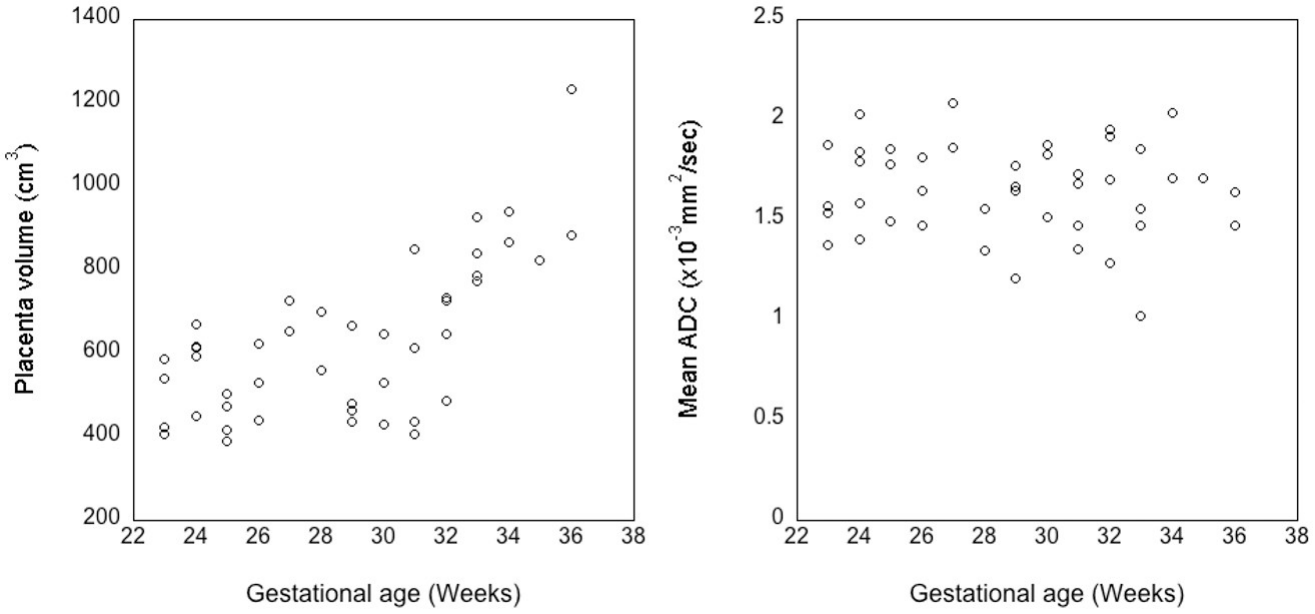
Placental volume of the imaging cohort showed the expected correlation between placental volume and gestational age (ρ = 0.62, p <0.0001). No significant correlation was observed between mean ADC value and gestational age (ρ = -0.06, p = 0.69).

Table 3 shows Spearman’s correlation coefficient for the Haralick texture features found to be significantly (q <0.05) associated with gestational age. Of the 13 textural features investigated, only 5 of them showed significant correlation, with at least one MRI acquisition (Table 3). Among the MR acquisitions, only the T2W (brain specific single slice), T1W & T2W (gravid uterus) features were associated with gestational age in the entire cohort. No significant correlations were observed between Haralick texture features of DW image, DWI-derived ADC map, T1W (breath-hold) image and gestational age. We investigated the SNR of DW image acquired at each b value. We found that SNR for the b = 0 s/mm^2^ was 32 and comparable to the average SNR of T1W (SNR = 20.3) and T2W (SNR = 38) images. The SNR of b = 800 s/mm^2^ images was 8, which was sufficiently high for reliable ADC map derivation [29,30]. No significant correlations were observed between mean ADC values and gestational age (Fig 4). Histogram normalization of DWI ROIs before texture extraction did not impact correlation with gestational age.

**Table 3:** Spearman correlation coefficient between the Haralick texture features that were found to be significant from placental MR acquisitions and gestational age. The corresponding q values (FDR adjusted p-values) are provided in brackets. Correlation coefficient values that were statistically significant are highlighted.

Acquisitions	f3 Correlation	f4 Sum of squares	f7 Sum variance	f8 Sum entropy	f9 Entropy
Entire Placenta T1W	0.42 (0.04)	0.35 (0.11)	0.36 (0.11)	0.35 (0.11)	0.47 (0.03)
Entire Placenta T2W	0.42 (0.04)	0.24 (0.24)	0.25 (0.23)	0.27 (0.19)	0.47 (0.03)
Single Slice T1W	0.33 (0.15)	0.1 (0.63)	0.14 (0.49)	0.07 (0.69)	-0.09(0.63)
Single Slice T2W	0.36 (0.11)	0.47 (0.03)	0.47 (0.03)	0.47 (0.03)	0.31 (0.18)
Single Slice DWI (b = 800 s/mm^2^)	-0.19 (0.34)	-0.25(0.23)	-0.26(0.23)	-0.25(0.24)	-0.2 (0.32)
Single Slice ADC map	0.11 (0.57)	0.14 (0.48)	0.14 (0.48)	0.34 (0.13)	0.29 (0.18)

## Discussion

In this paper, we investigated the feasibility of applying texture features based on second order statistics on retrospective clinical fetal MR images. We demonstrated that Haralick texture features, derived primarily from clinically acquired T1W and T2W MRI acquisitions, correlate with gestational age. The importance of taking into account gestational age specific textural changes was eluded in Dahdouh et al [31] where texture analysis of longitudinal T2W placental scans was used to study fetal growth restriction condition. Placental MR relaxation parameters such as T1, T2, and T2* have been shown to correlate with gestational age [32,33]. However, in clinical setting, longitudinal or prolonged acquisitions to generate quantitative T1, T2 maps are challenging for fetal MRI. Using gray scale routine fetal MR images that are clinically acquired, we are in essence defining the aging of the normal placenta with MR based on textural analysis. This will serve as a baseline for normal characteristics in order to compare diseased states, most importantly placental insufficiency and placental invasion. The heterogeneity significantly changes later in pregnancy as already described by ultrasound [6,34] and therefore the MR finding of invasion or insufficiency must be made in this context.

The MR findings of increased heterogeneity on textural analysis are based on the spatial relationship between pixels in an ROI. These variations in signal intensities are felt to represent aging of the cotyledon, the maternal fetal unit. On the macroscopic level, these changes in the cotyledon are manifest as better definition of the septa, and increased as well as decreased signal intensities of calcifications and fibrin deposition. On the physiologic level, hemodynamics, oxygenation, and exchange functions of the maternal-fetal unit are assumed to be constant. Therefore, it may be the physiology of tissue aging that is observed. The placental analysis differences between the total placenta volumes compared to single slice are likely because of the large differences in the size of the dataset. Clearly, a larger prospective placenta-specific study would enable a standardized approach to fetal placental imaging and address the differences.

For our study, we did not find a statistically significant correlation between gestational age and texture features of gravid uterus DWI and ADC ROIs or between gestational age and mean ADC placental values. Texture features derived from ROIs that went through intensity normalization and histogram equalization were investigated but neither improved the statistical outcome for DWI or ADC maps. We found a positive correlation between placental volume and gestational age, in agreement with existing literature [35]. It was previously thought that the relative ADC values were stable during gestation, an implication that water diffusion in the normal placental tissue is not affected by the amount of connective tissue or calcification, felt to represent the aging process [36]. However, Capuani et al [37] recently described a negative correlation between ADC values of placental ROIs and gestational age for GA ≥30 week. In our study, we did not observe this trend. The discrepancy may come from the difference in ROI coverage of the placenta. Our study measured the average ADC across the total placenta in the slice and we did not segment the placenta into 3 different groups, as their study did. Furthermore, Capuani et al. also used a bi-exponential fit on 7 b-values DW images to compute the ADC values, while our study was limited to the routine clinical DWI with 2 b-values acquisitions and the corresponding mono-exponential fitting for ADC derivation.

Second-order statistics was performed to measure how different gray levels are positioned relative to each other, essentially searching for heterogeneity. Use of second-order statistics was able to determine that directional anisotropy does not impact analysis, which emphasizes that planar orientation of images is not important [38]. Of all the functional textural measures, function 9 represents entropy, which is a measure of randomness. Therefore, it makes sense that increasing visual heterogeneity that we see in the MR images would correlate with randomness. The structural features that affect heterogeneity would include differences in contrast and organizational appearance of the placental cotyledons. Thus, characterization of the placenta based on Haralick texture features is potentially useful, evident in its growing application in the field of cancer so far.

Our study group did not include women with placental insufficiency or placental invasion. The MRI’s had normal fetal findings or only subtle increased measurement of the lateral ventricle of the central nervous system. Fetuses with isolated mild ventriculomegaly have an overall good prognosis. However, there are many functions of the placenta of which we are not yet aware, and may be found to be associated with the condition. This and the lack of outcomes are weaknesses of our study that should be acknowledged. The number of women included in this study is low and the result would require validation on a larger dataset. Nevertheless, we found in this small series that increasing heterogeneity of the placenta as the result of the aging process can be seen with MR texture analysis. Characterization of the placenta in abnormal states should therefore be performed in the context of fetal gestational and placental age.

Another limitation of present study is the small subset of texture features evaluated for our cohort. The Haralick texture features are the most classical of an ever-growing family of image descriptors. In addition to other statistical texture metrics that describe second-order image statistics, more recently developed texture metrics, such as syntactic, structural, or spectral texture features, could likewise be applied to the human placenta. In a recent application of texture analysis to fetal MR images, 15300 texture metrics per ROI were generated and analyzed [39]. It is not yet clear which texture metrics are optimal for characterization of the normal placenta and how best to avoid over-fitting and inherent redundancy [40]. We therefore sought to explore the thirteen classical Haralick texture features in this preliminary study as a proof-of-concept [40].

## Conclusions

From this retrospective study, we were able to confirm the increase in placental heterogeneity with gestational age by comparing Haralick texture features on MR images. Gestational age is the confounding variable for any type of placental analysis. The importance of this study is to identify baseline MR textural changes at certain gestational ages from which placental diseased states may be compared. Specifically, when evaluating for placental invasion or insufficiency, findings should be evaluated in the context of the placental aging process, which occur throughout gestation.
